# Quantitative Analysis of the Efficiency of Clonal Deletion in the Thymus

**DOI:** 10.1155/1994/92025

**Published:** 1994

**Authors:** Lisa M. Spain, Leslie J. Berg

**Affiliations:** Department of Cellular and Developmental Biology Harvard University 16 Divinity Avenue Cambridge Massachusetts 02138 USA

**Keywords:** Negative selection, thymic epithelium, T-cell receptor transgenic, thymix organ culture

## Abstract

One of the major mechanisms for establishing self-tolerance is the clonal deletion of self-reactive T cells during their development in the thymus. Using a TCR transgenic mouse model, we have established a quantitative *ex vivo* assay for examining the sensitivity and specificity of negative selection. Thymic organ cultures established from mice of varying MHC haplotypes were incubated with antigen, and the efficiency of clonal deletion assessed. We show here that clonal deletion of CD4^+^8^+^ thymocytes is sensitive to both the gene dosage and the allelic variation of MHC class II molecules expressed on thymic antigen-presenting cells. We also find that when epithelial cells in the thymic cortex are the only antigen-presenting cells expressing the appropriate MHC class II molecules, negative selection of CD4^+^8^+^ cells is as efficient as when antigen is presented on all thymic antigen-presenting cells. These studies demonstrate that the induction of self-tolerance via clonal deletion in the thymus is a function not only of antigen concentration, but also of MHC class II cell-surface density. In addition, together with the reports of others, these results confirm that cortical epithelial cells can mediate negative selection, and demonstrate that they do so in the intact thymic microenvironment.


*Developmental Immunology*, 1994, Vol 4, pp. 43–53
Reprints available directly from the publisher
Photocopying permitted by license only

© 1994 Harwood Academic Publishers GmbH
Printed in Singapore

### Quantitative Analysis of the Efficiency of Clonal Deletion in the Thymus

LISA M. SPAIN and LESLIE J. BERG*

*Department of Cellular and Developmental Biology, Harvard University, 16 Divinity Avenue, Cambridge, Massachusetts 02138*

One of the major mechanisms for establishing self-tolerance is the clonal deletion of self-reactive T cells during their development in the thymus. Using a TCR transgenic mouse model, we have established a quantitative *ex vivo* assay for examining the sensitivity and specificity of negative selection. Thymic organ cultures established from mice of varying MHC haplotypes were incubated with antigen, and the efficiency of clonal deletion assessed. We show here that clonal deletion of $CD4^+8^+$ thymocytes is sensitive to both the gene dosage and the allelic variation of MHC class II molecules expressed on thymic antigen-presenting cells. We also find that when epithelial cells in the thymic cortex are the only antigen-presenting cells expressing the appropriate MHC class II molecules, negative selection of $CD4^+8^+$ cells is as efficient as when antigen is presented on all thymic antigen-presenting cells. These studies demonstrate that the induction of self-tolerance via clonal deletion in the thymus is a function not only of antigen concentration, but also of MHC class II cell-surface density. In addition, together with the reports of others, these results confirm that cortical epithelial cells can mediate negative selection, and demonstrate that they do so in the intact thymic microenvironment.

KEYWORDS: Negative selection, thymic epithelium, T-cell receptor transgenic, thymix organ culture.

### INTRODUCTION

The analysis of thymic selection in T-cell receptor (TCR) transgenic mice has demonstrated that the specificity of the TCR determines the fate of immature thymocytes. Thymocytes bearing TCRs specific for self major histocompatibility complex (MHC) class I proteins differentiate into $CD8^+$ T cells (Kisielow et al., 1988b; Sha et al., 1988a, 1988b; Teh et al., 1988), whereas thymocytes bearing TCRs specific for self MHC class II proteins differentiate into $CD4^+$ T cells (Berg et al., 1989b; Kaye et al., 1989). These studies have also demonstrated that a thymocyte will die if its TCR cannot bind self MHC proteins, ensuring that all mature T cells are likely to recognize foreign peptides bound to self MHC molecules. Finally, studies of TCR transgenic as well as normal mice have demonstrated that any thymocytes that respond to self antigen expressed in the thymus are eliminated (negative selection) (Berg et al., 1989a; Kappler et al., 1987, 1988; Kisielow et al., 1988a; Sha et al., 1988a).

The variables controlling clonal deletion in the thymus have not been completely described. Previous studies examining heterogeneous TCRs have demonstrated that a threshold exists, below which concentrations of antigen are incapable of inducing clonal deletion (Schild et al., 1990). In addition, it has been shown that the extent of clonal deletion, for a single TCR, also depends on antigen concentration (Spain and Berg, 1992; Vasquez et al., 1992). However, no systematic examination of the effects of MHC sequence variability and surface density on negative selection of a single defined TCR has been described. Our previous studies using a TCR transgenic mouse system demonstrated that the efficiency of positive selection is profoundly influenced by alterations in both the surface density and the specific sequence of MHC molecules expressed in the thymus (Berg et al., 1990). These results suggested that the induction of self-tolerance via clonal deletion might also be sensitive to subtle alterations in the expression of MHC molecules.

*Corresponding author.

A second unresolved issue of T-cell development is how thymocytes are able to distinguish the MHC/peptide complexes inducing positive selection from those inducing negative selection. One possibility is that the affinity of the TCR for the MHC/peptide ligands encountered in the thymus is the sole determinant of thymic selection. Alternatively, it has been suggested that different antigen-presenting stromal cells of the thymus may be specialized in their ability to mediate these two selection events by providing specific costimulatory interactions. (Lo and Sprent, 1986; Marrack et al., 1988, Benoist and Mathis, 1989; Berg et al., 1989). In addition, we and others (Lo and Sprent, 1986; Benoist and Mathis, 1989; Berg et al., 1989) have previously shown that the appropriate MHC class II expression on thymic cortical epithelium is both necessary and sufficient to induce positive selection. Recent studies have demonstrated that thymic epithelium can also mediate negative selection (Carlow et al., 1992; Iwabuchi et al., 1992; Speiser et al., 1992), indicating that these cells are not specialized for positive selection. However, the relative efficiency of cortical epithelial cells for inducing clonal deletion was not addressed in these prior studies due to an inability to control the amount of antigen available to the MHC molecules on the epithelium. Thus, it remained possible that negative selection by antigens present on thymic epithelium could be physiologically important only for very abundant or high-affinity antigens.

Here we have used a quantitative assay, based on thymic organ cultures derived from TCR transgenic mice, to test the influence of MHC surface density and sequence variability on the efficiency of clonal deletion. In addition, we have directly compared the efficiency of clonal deletion mediated by peptides presented solely on thymic cortical epithelium to that of peptides presented on the total population of thymic antigen-presenting cells. We find that in addition to peptide concentration, both the surface density and specificity of the MHC class II molecules have dramatic effects on the efficiency of clonal deletion. Furthermore, we find that peptides presented on thymic cortical epithelial cells induce clonal deletion as efficiency as peptides presented on any other thymic cell type. These results demonstrate that variables other than antigen concentration, including MHC surface density and specificity, have profound effects on the efficiency of clonal deletion in the thymus.

### RESULTS

### The Efficiency of Clonal Deletion in Newborn Thymic Organ Cultures Depends on MHC Sequence and Surface Density

To quantitatively assess the efficiency of clonal deletion in the thymus, we have used a thymic organ culture assay derived from TCR transgenic mice. For these studies, we used mice transgenic for the α and β chains of the 2B4 T-cell receptor (TCR), which is specific for MHC class II I-$E^k$ plus the *c*-terminal fragment of moth or pigeon cytochrome *c*. These transgenic mice have been extensively studied and have been shown to express the α and β chains of the 2B4 TCR on <95% of thymocytes (Berg et al., 1989b). In addition, we found that thymocytes bearing the 2B4 TCR develop into $CD4^+$ T cells only when I-$E^k$, or the closely related hybrid molecule $E^k_\alpha K^b_\beta$, is expressed in the thymus (Berg et al., 1989b). Our previous studies have shown that fetal or neonatal organ cultures of 2B4 TCR transgenic thymi are an effective method for the analysis of antigen-mediated deletion (Spain and Berg, 1992). These studies demonstrated that when moth cytochrome *c* peptide 88-103 (MCC) is added to the culture medium of 2B4 transgenic thymuses, $CD4^+8^+$ thymocytes are efficiently eliminated. MCC peptide had no effect on organ cultures established from nontransgenic thymuses or on I-$E^-$(H-$2^b$)2B4 TCR transgenic thymuses. Furthermore, control I-E binding peptides from hemoglobin (βd minor, residues 67–76) or hen egg lysozyme (residues 85–96) also did not induce clonal deletion of 2B4 transgenic $CD4^+8^+$ thymocytes (Spain and Berg, 1992; and data not shown).

We first examined the effects of varying MHC class II I-E surface density and allele specificity on negative selection. For these experiments, we compared clonal deletion in 2B4 TCR transgenic mice congenic for three different MHC haplotypes (see Table 1). H-$2^k$ mice express a high density of I-$E^k$ on all antigen-presenting cells. H-$2^{kxb}$ mice, which have only one functional copy of the $E_\alpha$ gene

TABLE 1
B10 Haplotype Designations at the I-E Locus

| Strain | $E_\alpha$ alleles | $E_\beta$ alleles |
|---|---|---|
| H-$2^k$ | k;k | k;k |
| H-$2^{kxb}$ | k;– | k;b |
| H-$2^{bxi5}$ | k;– | b;b |
| H-$2^{bxh4}$ | –;– | b;k |
| H-$2^b$ (ΔY transgene) | –;– (k transgene) | b;b |

(Mathis et al., 1983), express significantly lower levels of surface I-E; furthermore, this I-E is a mixture of I-$E^k$ and the hybrid I-E molecule, $E^k_\alpha E^b_\beta$. Finally, H-$2^{bxi5}$ mice, which also have only one functional copy of the $E_\alpha$ gene, express only low levels of the hybrid $E^k_\alpha E^b_\beta$ molecule on their antigen-presenting cells (Berg et al., 1990). We have previously shown that the efficiency of positive selection of 2B4$^+$CD4$^+$ thymocytes varies dramatically between mice of these three different MHC haplotypes, with H-$2^k$ being the most efficient and H-$2^{bxi5}$ the least efficient (Berg et al., 1990).

To examine the effects of MHC variation on negative selection, neonatal thymuses from 2B4 TCR transgenic mice of each type were cut into 6 pieces and incubated at the air-medium interface in the presence of variable concentrations of peptides. After 2 days, the cultured lobes were dissociated and stained with antibodies against CD4, CD8, and 2B4 TCR. Stained cells were pregated for 2B4 TCR α-chain expression (of which <95% also expressed the 2B4 β chain) and further analyzed for CD4 and CD8 expression. Results for two individual mice, one of the H-$2^k$ and one of the H-$2^{kxb}$ haplotype, are shown in Fig. 1(A). Thymic fragments were incubated with no peptide, 0.01 μM, or 0.1 μM moth cytochrome *c* (MCC) peptide. As expected, CD4$^+$8$^+$ thymocytes were deleted by MCC peptide in a dose-dependent manner. Furthermore, thymocytes in the H-$2^k$ thymus were more sensitive to deletion by the same concentration of MCC peptide than were those in the H-$2^{kxb}$ thymus. A summary of the data from a more extensive peptide titration indicates that approximately tenfold more MCC peptide was required to induce an equivalent level of deletion in H-$2^{kxb}$ than H-$2^k$ thymuses (Fig. 2). Interestingly, clonal deletion mediated by the MCC peptide was even more inefficient in 2B4 TCR transgenic mice of the H-$2^{bxi5}$ haplotype. We found that when MCC peptide is presented on the hybrid $E^k_\alpha/E^b_\beta$ molecule approximately 100-fold higher concentrations of peptide are required to induce an equivalent level of deletion as in H-$2^k$ mice (Fig. 2).

The 2B4 hybridoma was originally generated from an H-$2^k$ mouse immunized with pigeon cytochrome *c* (Hedrick et al., 1982). As with most I-$E^k$/cytochorme *c* reactive T cells, the 2B4 hybridoma responds better to the homologous peptide from moth cytochrome *c* than to the original immunogen, pigeon cytochrome *c* (Hedrick et al., 1982; Fox et al., 1987). Thymic organ cultures derived from H-$2^{kxb}$ 2B4 TCR transgenic mice were incubated with a range of concentrations of the pigeon cytochrome *c* (PCC) peptide, and the elimination of CD4$^+$8$^+$ thymocytes was examined after 48 hr. As can be seen in Fig. 2, approximately tenfold higher concentrations of PCC peptide than MCC peptide are required to induce the same extent of clonal deletion.

### The Efficiency of Negative Selection by Cortical Epithelium Is Comparable to that Induced by Total Thymic-Presenting Cells

In order to determine the efficiency with which thymic cortical epithelial cells can mediate negative selection, the exposure of developing thymocytes to "self" antigen must be controlled both spatially and temporally. To do this, we used a second line of transgenic mice that carry an MHC class II $E^k_\alpha$ gene in their germline. Mice of the H-$2^b$ MHC haplotype lack all surface I-E expression due to a deletion in the promoter region of the endogenous $E_\alpha$ gene (Mathis et al., 1983). Surface I-E expression in these mice can be restored with a transgenic $E^k_\alpha$ gene. The ΔY line of transgenic mice carries an $E^k_\alpha$ gene with a small deletion in the promoter region; in mice, this mutated $E^k_\alpha$ gene leads to I-E expression in the thymus almost exclusively on cortical epithelial cells, although ~1% of medullary dendritic cells are also positive (van Ewijk et al., 1988). Using the 2B4/ΔY double transgenic mice, we have previously shown that I-E expression on thymic cortical epithelium is both necessary and sufficient for positive selection of the 2B4 TCR (Berg et al., 1989b). In those experiments, 2B4$^+$CD4$^+$T cells developed in ΔY mice, but not in mice lacking I-E or expressing it only on stromal cells of the thymic medulla (Berg et al., 1989b).

We then tested neonatal thymic organ cultures derived from the 2B4/ΔY double transgenic mice for clonal deletion in the presence of MCC peptide. No deletion of CD4$^+$8$^+$ thymocytes was detected at any peptide concentration (data not shown). Immunohistochemistry of cryosections from fetal and neonatal ΔY thymuses indicated that surface I-E expression was completely lacking at these stages (data not shown). We determined that ΔY mice >6 weeks old showed levels of cortical epithelial I-E expression comparable to that of H-$2^k$ mice, as has been previously reported (van Ewijk et al., 1988). The lack of I-E expression in neonatal mice required us to modify the thymic organ culture technique for adult thymuses. Adult thymuses were isolated, cut

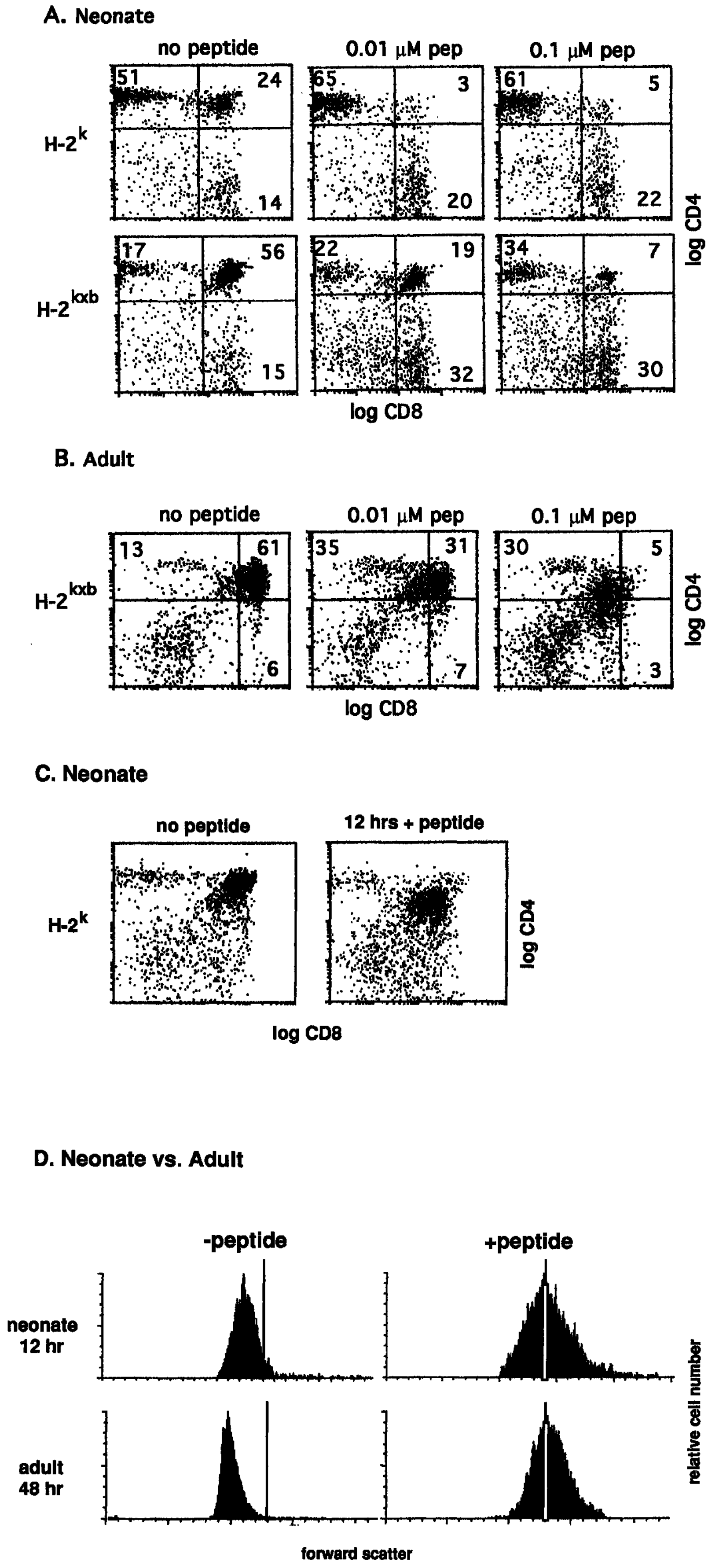

FIGURE 1. Effects of antigenic peptide on neonatal and adult 2B4 transgenic thymic organ cultures. Thymic fragments from (A) neonatal $H\text{-}2^k$ and $H\text{-}2^{kxb}$ mice and (B) adult $H\text{-}2^{kxb}$ mice were incubated in the indicated concentrations of MCC (88–103) peptide for 48 hr. The fragments were dissociated and stained for 2B4 α, CD4, and CD8. The samples were gated for expression of 2B4 α and the results for CD4 versus CD8 expression is displayed. The percentage of cells in each quadrant is indicated. (C) Neonatal thymic fragments from $H\text{-}2^k$ mice were incubated in the absence (left) or presence (right) of 1 μM MCC peptide for 12 hr and stained as before. (D) Forward-scatter profiles of $CD4^+8^+/CD4^{low}8^{low}$ thymocytes cultured in the absence (left) or presence (right) of 1-μM peptide for the indicated time. Profiles for neonatal thymic organ cultures are shown above, and adult thymic organ cultures below. A vertical line is shown to provide a reference point in comparing the forward-scatter profiles.

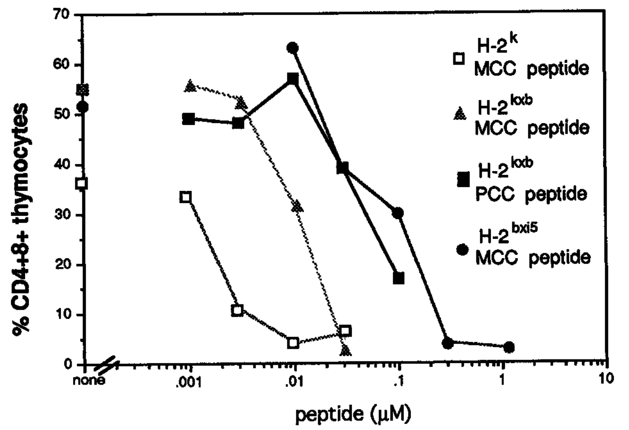

FIGURE 2. MHC allelic variation and antigenic peptide sequence alter the dose response of clonal deletion in neonatal thymic organ culture. The α and β 2B4 TCR transgenes were crossed onto different B10 congenic MHC haplotypes as indicated (see Table 1). Due to a deletion in the promoter of the $E_{\alpha}$ gene in H-$2^{b}$ mice, H-$2^{kxb}$ express approximately one-half as much surface I-E as do homozygous H-$2^{k}$ mice; in addition, H-$2^{kxb}$ mice express both I-$E^{k}$ and the hybrid $E^{k}_{\alpha}/E^{b}_{\beta}$ molecule. H-$2^{bxi5}$ mice (derived by crossing 2B4$^{+}$ H-$2^{b}$ mice to the B10.A(5R) strain, see Table 1) express heterozygous levels of only the $E^{k}_{\alpha}/E^{b}_{\beta}$ hybrid molecule. Neonatal thymic fragments were incubated for 48 hr in the indicated concentrations of either moth or pigeon cytochrome *c* peptides, and analyzed by FACS as described in Materials and Methods. The percentages of CD4$^{+}$8$^{+}$ thymocytes as a function of peptide concentration are displayed.

into small pieces (~1 mm$^{3}$), and placed in culture floating on sterile sponges. We determined that individual ~1 mm$^{3}$ thymic fragments contained reasonably equivalent percentages of thymocyte subsets. For example, 6 fragments from a 2B4 transgenic H-$2^{k}$ thymus contained on average (+/− standard deviations) 16.3% ± 6.5% CD4$^{+}$8$^{-}$ thymocytes, 62.2% ± 10.1% CD4$^{+}$8$^{+}$ thymocytes, 14.2% ± 6.7% CD4$^{-}$8$^{-}$ thymocytes, and 7.2% ± 2.4% CD4$^{-}$8$^{+}$ thymocytes after 2 days in organ culture.

Thymic fragments from age-matched 2B4 transgenic mice of either the H-$2^{kxb}$ or the ΔY genotypes were incubated with dilutions of moth cytochrome *c* peptide, and the extent of deletion of CD4$^{+}$8$^{+}$ thymocytes was determined by antibody staining. The results of a representative CD4 and CD8 staining (previously gated on 2B4 TCRα$^{+}$ cells) of adult H-$2^{kxb}$ thymic fragments incubated with no peptide, 0.01 μM, and 0.1 μM are shown in Fig. 1(B). We found that peptide added to adult thymic fragments resulted in a reduction in surface staining of CD4 and CD8, rather than in complete deletion as was observed in the neonatal samples; compare Figs. 1(A) and 1(B). Thymocytes with down-modulated CD4 and CD8 (CD4$^{low}$8$^{low}$) persisted in the thymic organ cultures for at least 72 hr after peptide addition (the latest time point tested). In addition, we found that incubation in the presence of peptide did not reduce the number of thymocytes recovered from the organ cultures. Because we have previously reported that deletion of CD4$^{+}$8$^{+}$ thymocytes in neonatal organ cultures is preceded by a stage during which the thymocytes exhibited reduced CD4 and CD8 expression (Spain and Berg, 1992; and see Fig. 1(C), we decided to determine whether CD4$^{low}$8$^{low}$ thymocytes present in adult thymic organ cultures were in other respects similar to neonatal thymic clonal deletion intermediates. We compared forward- and side-scatter parameters of CD4$^{low}$8$^{low}$ thymocytes at an early time point (12 hr) in neonatal cultures with CD4$^{low}$8$^{low}$ thymocytes from adult organ cultures. As shown in Fig. 1(D), CD4$^{low}$8$^{low}$ thymocytes in both cases exhihit a higher degree of forward light scatter indicative of increased size. Thymocytes from neonates and adults also had increased side scatter following peptide treatment (data not shown). Based on these results, we think it likely that CD4$^{low}$8$^{low}$ thymocytes in adult organ cultures are in fact intermediates in the process of clonal deletion, but that for unknown reasons, perhaps because of age or size-related differences in thymic architecture, the adult thymuses are unable to support full deletion during the 48-hr culture period. Others have also reported that CD4 and CD8 down-regulation occurs as an intermediate in the deletion process (Carlow et al., 1992; Iwabuchi et al., 1992; Swat et al., 1992). Page and co-workers (1993) further report that CD4$^{low}$8$^{low}$ thymocytes can be induced to undergo apoptosis (in the absence of further antigenic stimulation) by class II transfected L929 fibroblasts or spleen APCs *in vitro*.

Our data show that the peptide sensitivity of the adult thymocytes (manifested as a decrease in CD4$^{hi}$CD8$^{hi}$ thymocytes) is absolutely consistent with the sensitivity of deletion in neonatal thymic organ cultures; compare Figs. 1(A) and 1(B) for H-$2^{kxb}$ mice. We therefore calculated the extent of "deletion" in the adult cultures as the reduction in the percent of CD4$^{hi}$8$^{hi}$ thymocytes in the peptide-treated compared to the untreated control thymi. The results of a representative experiment (one of four independent trials) are shown in Table 2. In both the ΔY and the control H-$2^{kxb}$ thymuses, significant depletion of CD4$^{hi}$8$^{hi}$ thymocytes was observed at MCC peptide concentrations down to 0.01 μM. Most importantly, the ΔY and control

TABLE 2
Titration of MCC Peptide-Mediated CD4/CD8 Down-regulation in Adult 2B4 Transgenic. (H-$2^{kxb}$), 2B4/ΔY Transgenic, and Non-transgenic Thymuses[a]

| | Fraction of $CD4^+8^+$ cells | | |
|---|---|---|---|
| MCC peptide (μM) | H-$2^{kxb}$ | ΔY | Non transgenic |
| 0 | 1 | 1 | 1 |
| 0.01 | 0.5 | 0.4 | |
| 0.1 | 0.08 | 0.06 | |
| 1 | 0.04 | 0.01 | |
| 4 | 0.04 | 0.01 | |
| 10 | 0.03 | 0.003 | 1.05 |
| 40 | 0.03 | 0.001 | |

[a] Thymic fragments were incubated in organ culture with the indicated concentration of MCC peptide. After 2 days, the fragments were dissociated and the cells simultaneously stained for 2B4 α, CD4, and CD8 expression, and analyzed by FACS. The fraction of 2B4 $\alpha^+CD4^+8^+$ (as opposed to $CD4^{low}8^{low}$) thymocytes present in peptide-treated relative to untreated thymic fragments is shown for each sample.

thymic fragments did not differ in their efficiencies of mediating $CD4^+8^+$ down-regulation. The MCC peptide had no effect on thymocytes in nontransgenic thymic organ cultures (Table 2); in addition, non-MCC, I-E-binding peptides derived from hen egg lysozyme or hemoglobin also had no effect on transgenic thymic organ cultures (data not shown).

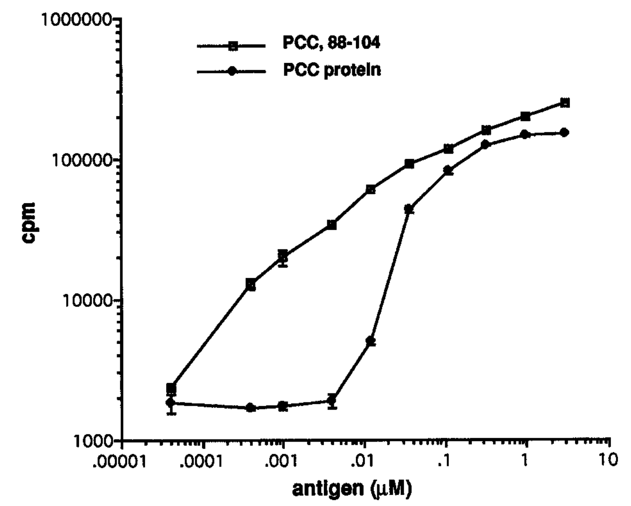

FIGURE 3. Synthetic PCC peptide is a more potent antigen for $2B4^+$ T cells than whole cytochrome *c* protein. 2B4 transgenic lymph node cells were stimulated with H-$2^k$ spleen antigen-presenting cells in the presence of the indicated concentrations of antigen. Each data point is the average proliferative response (± standard deviation) from triplicate wells. The experiment was repeated twice with identical results.

### Cortical Epithelial Cells Efficiently Process Whole Cytochrome *C* Antigen

Cortical epithelial cells have been found to be deficient in antigen processing and presentation for some intact antigens (Lorenz and Allen, 1989; Ransom et al., 1991). Consequently, we were interested in determining whether thymic cortical epithelial cells could process intact protein antigens as well as present synthetic peptide fragments. We expected that the whole protein cytochrome *c* might be a less potent antigen than synthetic peptide due to the extra requirements of proteolytic processing. In order to be able to account for this difference, we first tested the efficiency with which normal spleen antigen-presenting cells process and present pigeon cytochrome *c* protein as compared with synthetic peptide. Lymph node cells from a 2B4 TCR transgenic mouse were incubated with irradiated spleen cells and various concentrations of whole cytochrome *c* protein or peptide (PCC, 88–104) antigen. After 48 hr, cultures were pulsed with $^3H$-thymidine as a measure of proliferation. As shown in Fig. 3, we found that spleen cells do efficiently process whole cytochrome *c*, although the protein is a less efficient antigen on a molar basis than the synthetic peptide, as has previously been reported (Matis et al., 1983; Wettstein et al., 1991).

Because ΔY (H-$2^b$) mice express an $E^k_\alpha/E^b_\beta$ hybrid I-E protein that does not bind pigeon cytochrome *c*, the 2B4/ΔY double transgenic mice were crossed to the B10.A(4R) congenic strain that carries an $E^k_\beta$ gene together with the nonfunctional $E^b_\alpha$ allele. The resulting ΔY (H-$2^{bxh4}$) mice (see Table 1) express the I-$E^k$ MHC protein, which binds the pigeon cytochrome *c* peptide; in addition, the surface I-$E^k$ expression in these mice is controlled by the ΔY transgene. The sensitivity of $CD4^+8^+$ down-regulation was assessed by exposing thymic fragments in organ culture to different concentrations of pigeon cytochrome *c* whole protein versus synthetic peptide. As was the case in the proliferation assays (see Fig. 3), cytochrome *c* protein was less efficient than synthetic peptide at mediating down-regulation in both ΔY and control H-$2^k$ 2B4 transgenic thymus fragments (Fig. 4). However, at concentrations of 1 μM and higher, whole cytochrome *c* protein induced an equivalent effect on $CD4^{hi}CD8^{hi}$ cells in both ΔY and H-$2^k$ thymuses (Fig. 4). These results suggest that cortical epithelial cells are capable of processing and presenting antigen to thymocytes. We also reproducibly observed a slight decrease in the sensitivity of $CD4^+8^+$ cells in ΔY thymuses compared to H-$2^k$ thymuses when incubated with 0.1 μM cytochrome *c* protein, suggesting that cortical epithelial cells may be somewhat less effective at processing antigen.

### DISCUSSION

We have examined several factors influencing the outcome of clonal deletion in the thymus. Our analysis has focused on a single TCR specificity in order to examine the effects of antigen concentration, MHC surface density, and MHC allele specificity on the efficiency of negative selection. The fine specificity of the 2B4 TCR has been characterized in great detail (Hedrick et al., 1982). The 2B4 hybridoma was generated by immunizing an H-$2^a$ (I-$E^k$-positive) mouse with pigeon cytochrome *c*. However, in addition to responding to PCC + I-$E^k$, this hybridoma also responds to MCC+I-$E^k$. In fact, about tenfold lower concentrations of MCC peptide than PCC peptide are required to elicit the same level of resposne. Additionally, this hybridoma cross-reacts on MCC plus the hybrid I-E molecule, $E^k_\alpha/E^b_\beta$, although the response to this ligand also requires about tenfold more peptide to elicit an equivalent response (Fink et al., 1986). Interestingly, clonal deletion of $2B4^+$ $CD4^+8^+$ thymocytes follows exactly the same pattern. Approximately tenfold more PCC peptide than MCC peptide is required to induce the same level of deletion of $2B4^+$ thymocytes; furthermore, clonal deletion mediated by MCC + $E^k_\alpha/E^b_\beta$ is approximately ten- to 100-fold less efficient than deletion mediated by MCC + I-$E^k$.

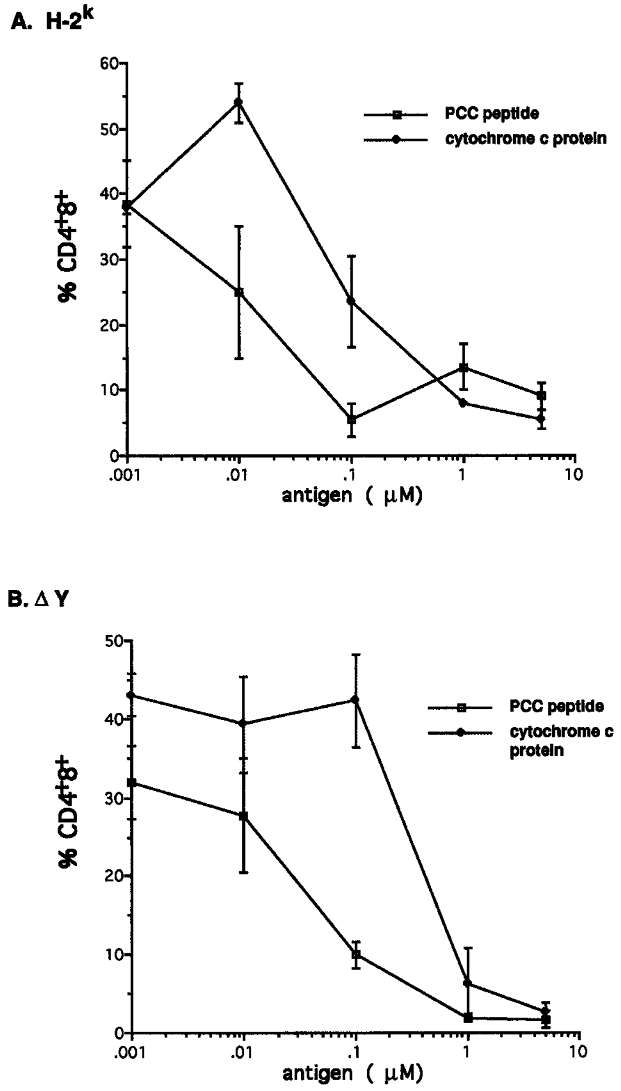

FIGURE 4. Clonal deletion by synthetic PCC peptide versus intact cytochrome *c* protein. Fragments from (A) 2B4 transgenic, H-$2^k$ and (B) 2B4/ΔY transgenic, H-$2^{bxh4}$ thymuses (see Table 1 for strain designations) were incubated in organ culture with varying concentrations of antigen. After 48 hr, thymic fragments were dissociated and the cells simultaneously stained for 2B4 α, CD4, and CD8 expression, and analyzed by FACS as described in Materials and Methods. The percentage of $2B4^+$, $CD4^+8^+$ thymocytes in thymic fragments is shown for each concentration of antigen. Open squares indicate values for synthetic PCC peptide; closed diamonds indicate values for whole cytochrome *c* protein.

In addition, we find that MHC surface density and MHC allele specificity also play a significant role in determining the efficiency of clonal deletion. When antigen-presenting cells carry one nonfunctional $E_\alpha$ allele, as in H-$2^{kxb}$ mice, they express ~50% as much total I-E as homozygous H-$2^k$ cells (Berg et al., 1990); in addition, H-$2^{kxb}$ mice express both $E^k_\alpha/E^b_\beta$ and $E^k_\alpha/E^k_\beta$. We show that approximately tenfold higher levels of MCC peptide are required to induce the same degree of clonal deletion in H-$2^{kxb}$ thymuses as is observed in homozygous H-$2^k$ thymuses (Fig. 2). We also found tht heterozygous levels of $E^k_\alpha E^k_\beta$ alone required an additional ten fold higher concentration of MCC peptide to induce the same degree of deletion (Fig. 2). Had the lower level of $E^k_\alpha E^k_\beta$ present in H-$2^{kxb}$ thymuses been capable of inducing deletion with an efficiency equivalent to that in the homozygous H-$2^k$ thymuses, we would have seen similar peptide sensitivity in the two cases, regardless of the presence of $E^k_\alpha E^b_\beta$. Instead, the results from H-$2^{kxb}$ were immediately between the H-$2^{bxi5}$ (heterozygous levels of $E^k_\alpha E^k_\beta$) and H-$2^k$ haplotypes, demonstrating that both allele type and MHC density play a role in modulating deletion sensitivity. However, our data do not address which of these two variables is more important in modulating deletion sensitivity.

Our previous studies indicated that positive selection is equally sensitive to the surface density of the selecting MHC molecules. For instance, twice as many mature 2B4$^+$CD4$^+$T cells are generated in homozygous H-2$^k$ mice as in heterozygous H-2$^{kxb}$ mice (Berg et al., 1990). In this regard, clonal deletion is much more similar to positive selection than it is to activation, because activation of the 2B4 hybridoma is not sensitive to this same difference in MHC surface density (Hedrick et al., 1982). In fact, primary *in vitro* stimulation of T cells from cytochrome *c*-primed mice (Matis et al., 1982a, 1983) as well as stimulation of long-term cytochrome *c*-reactive T-cell lines (Matis et al., 1982b) are also not affected by these changes in I-E surface density.

Taken together, these data indicate that clonal deletion in the thymus is not absolute. For thymocytes of a given specificity developing in the presence of their known MHC/peptide ligand, clonal deletion may eliminate some cells, whereas others of the same specificity survive and mature. Furthermore, it is not simply the concentration of antigen that influences this outcome, but the surface density of MHC molecules as well. In each case, a relatively wide range of antigen concentrations (over approximately tenfold) results in the intermediate state of partial deletion of reactive thymocytes. Presumably, in such a situation self-reactive thymocytes that survive development in the thymus would be exported to the periphery. These results attest to the need for a mechanism of peripheral tolerance, as concentrations of antigen clearly capable of inducing a signal do not always succeed in inducing deletion of all thymocytes of the appropriate specificity.

These experiments also provide a quantitative measure of the efficiency of negative selection mediated by thymic epithelium in an *ex vivo* (thymic organ culture) system. By comparing ΔY vs. H-2$^{kxb}$ thymuses, we tested deletion in the absence and presence of I-E$^+$ thymic hemopoietic cells in two strains that were similar with regard to their efficiency of positive selection (Berg, 1989b). Had hematopoietic-derived cells been very much more efficient at deletion than epithelial cells, we would have expected to find that normal H-2$^{kxb}$ thymuses were significantly better at deletion than the I-E$^+$ hemopoietic cell-deficient ΔY strain. This prediction depends on the assumption that the level of I-E expression on epithelial cells, and the relative ability of I-E to present antigen, is not significantly higher in ΔY than in H-2$^{kxb}$ thymuses. Our experiments (unpublished observations) and those of others (van Ewijk et al., 1988) indicate that the level of I-E expression in the ΔY cortex is comparable to that in wild-type H-2$^k$ mice, although these observations, derived from immunofluorescence assays, may not show small differences in expression. In addition, in the ΔY mice, the I-E is composed entirely of $E^k_\alpha E^b_\beta$ hybrid molecules, whereas in the H-2$^{kxb}$ mice it is comprised of both $E^k_\alpha E^b_\beta$. Thus, although MHC density might be somewhat lower in H-2$^{kxb}$ versus ΔY thymi, the presence of the $E_\beta$ allele recognized more efficiently by the 2B4 TCR ($E^k_\beta$) is likely to compensate for a lower level of expression in H-2$^{kxb}$ thymuses. In fact, this conclusion is supported by the data on positive selection in these two strains. We have previously shown that positive selection occurs on thymic epithelial cells, and is very sensitive to I-E allele and gene dosage (Berg et al., 1990). When the efficiency of positive selection in ΔY and H-2$^{kxb}$ 2B4 transgenic mice is examined, we find that these two strains are directly comparable (Berg et al., 1989b).

Others have recently reported that thymic epithelial cells can induce clonal deletion of CD4$^+$8$^+$ thymocytes *in vivo* (Carlow et al., 1992; Speiser et al., 1992). Our experiments support and extend these results by strongly suggesting that thymic cortical epithelium is as efficient as any other thymic cell type at mediating peptide-specific negative selection of CD4$^+$8$^+$ thymocytes. Our conclusion is based on earlier experiments that ΔY thymuses do express I-E on a small percentage (1%) of medullary stromal cells (van Ewijk et al., 1988). It is unlikely that ΔY thymuses would be able to induce negative selection of CD4$^+$8$^+$ thymocytes as efficiently as wild type thymuses if this small subset of cells were solely responsible for clonal deletion. Furthermore, the medullary location of these cells would make them unlikely to effect the CD4$^+$8$^+$ thymocytes that reside in, and are eliminated in (Murphy et al., 1990), the thymic cortex.

Our results are inconsistent with reports that thymic epithelial cells are less efficient than other thymic stromal cells at deleting thymocytes (von Boehmer and Hafen, 1986; von Boehmer and Schubiger, 1984; Carlow et al., 1992). One possible explanation for this discrepancy might be that differences in the expression or accessibility of the deleting antigen could not be controlled in these previous studies. For instance, the higher efficiency of deletion of H-Y reactive T cells by bone marrow–derived versus epithelial cells might be due to higher expression of the H-Y antigen in bone marrow cells (Carlow et al., 1992). In a recent study, Jenkinson and co-workers (1992) have shown in an *in vitro* reconstituted thymic system that epithelial cells mediate deletion by SEB with lowered efficiency. It is possible that our results differ from those of Jenkinson and colleagues due to differences in superantigen versus convential antigen-mediated deletion.

It is also possible that differences between our results and those of others may be due to differences in antigen processing and presentation by thymic epithelium. To address this possibility, we determined the ability of thymic epithelium to process protein antigens. Our studies indicate that thymic cortical epithelial cells are capable of processing as well as presenting protein antigen to thymocytes undergoing selection in the thymus. The thymic cortical epithelial cells in ΔY mice were observed to be only slightly less efficient than other antigen-presenting cells at processing whole cytochrome *c* protein for negative selection. It has been previously reported that thymic epithelial cell lines may be deficient in the processing of some ovalbumin antigens (Ransom et al., 1991). Our results are consistent with the idea that thymic epithelium is able to present antigens, but that for some antigens, protein processing may be inefficient. Alternatively, we have not ruled out the possibility that other thymic cells could process the cytochrome *c* antigen and deliver peptides to the epithelial cells for presentation to thymocytes.

We also report here the unexpected finding that deletion occurs with slower kinetics in organ cultures derived from adult versus neonatal thymus. In exploring this phenomenon, we have further characterized the $CD4^{low}8^{low}$ deletion intermediates observed in neonatal thymic organ cultures and have found they have increased in size and density as measured by light-scatter parameters. One implication of our observations is that deletion might occur in two discrete steps, the first involving coreceptor down-regulation, and the second resulting in overt cell death and apoptosis. Evidence for a two-step mechanism for deletion of thymocytes *in vitro* has recently been reported (Page et al., 1993). In further support of the two-step hypothesis, we have also observed that chinese hamster ovary (CHO) fibroblasts (transfected with I-$E^k$) induce peptide-dependent coreceptor down-regulation and size increases without inducing cell death when cocultured with thymocytes (Spain and Berg, unpublished observations). We presume that CHO fibroblasts lack a cofactor essential for deletion, and suggest that adult thymic fragments in organ culture might also be limited in this presumed cofactor. This hypothesis, although speculative, is amenable to further experimentation.

From these experiments, we conclude that the efficiency of clonal deletion is influenced by MHC surface density as well as antigen concentration and MHC allele specificity. These data argue that TCR recognition of MHC/peptide ligands during positive and negative selection is strikingly similar. Furthermore, we find that both positive and negative selection can result from thymocyte interactions with the same antigen-presenting cells. These results indicate that thymic cortical epithelial cells are not specialized for the sole function of mediating positive selection, and suggest that the outcome of thymic selection is solely dependent on the specific details of the TCR interaction with MHC/peptide complexes.

### MATERIALS AND METHODS

### Mice

2B4 transgenic, ΔY transgenic (provided by D. Mathis and C. Benoist), and nontransgenic B10.BR/SgSnJ and B10.A(4R), B10.A(5R) (obtained from Jackson Labs) mice were maintained and bred under standard conditions.

### Thymic Organ Cultures

Neonatal thymuses were cultured according to procedures previously described (Spain and Berg, 1992). Adult and neonatal lobes were cut using small curved scissors into 1-$mm^3$ pieces and placed on a strip of nitrocellulose supported by a sterile gelatin sponge (Upjohn) soaked in RPMI/10% fetal calf serum plus $5\times10^{-5}$ M β-mercaptoethanol, gentamycin, penicillin, and streptomycin. Each experiment was repeated at least 3 times (unless otherwise noted in the figure captions), and a representative example given in the figures.

### Antigens

Cytochrome *c*, C-terminal peptides from moth (88–103, MCC) and pigeon (88–104, PCC) were synthesized by the Microchemistry Facility, Harvard University. Purity was analyzed by mass spectrometry and analytical HPLC. The MCC peptide was further purified by preparative HPLC. Whole pigeon cytochrome *c* (Sigma) was purified by HPLC. Antigen concentrations were confirmed by absorbance at 280 nm.

### FACS Analysis

Directly conjugated rat anti-mouse CD4-PE and directly conjugated rat anti-mouse CD8-Red 613 monoclonal antibodies were purchased from GIBCO-BRL. Other antibodies used were hamster anti-mouse CD3 (clone 500A2) Havran, et al., 1987 biotinylated anti-Vβ3 (clone KJ25) Pullen, et al, 1988 and FITC-conjugated anti-2B4 α chain (clone A2B4-2) (Samelson et al., 1983). FITC-conjugated goat anti-hamster IgG affinity-purified antibody was purchased from Caltag. Cultured thymic organs were dissociated and the cells stained using the earlier reagents for CD4, CD8, and CD3 simultaneously, and in some cases, parallel samples were stained for CD4, Vβ3, and the clonotypic 2B4 α chain. Flow cytometric analysis was performed on a FACScan instrument (Becton-Dickinson). Ungated data (10,000 events per sample) were collected in list mode using FACScan Research software and analyzed using LYSYS software (Becton-Dickinson). Before analysis of antibody staining, samples were gated on live cells based on forward- and side-scatter parameters.

### Antigenic Stimulation

$10^6$ irradiated B10.BR (I-$E^k$) spleen cells were plated with $10^5$ 2B4 transgenic lymph node cells in triplicate wells. After 48 hr, each well was labeled for 16 hr with $^3$H-thymidine (I μCi/well), harvested on filter discs (Skatron), and counted in Biofluor (DuPont).

### ACKNOWLEDGEMENTS

We thank M. Davis for peptides, C. Benoist and D. Mathis for the ΔY mice, and S. D. Heyeck and C. Gurniak for comments on the manuscript. This work was supported by the American Cancer Society, Inc., Grant #1M-654, and by a Cancer Research Institute/Florence and Edgar Leslie Charitable Trust Investigator Award to L.J.B. L.M.S. was supported in part by an NIH postdoctoral fellowship.

*(Received September 9, 1993)*

*(Accepted December 27, 1993)*

### REFERENCES

Benoist C., and Mathis D. (1989). Positive selection of the T cell repertoire: Where and when does it occur? Cell **58**: 1027–1033.

Berg L.J., Fazekas de St. Groth B., Pullen A.M., and Davis M.M. (1989a). Phenotypic differences between αβ and β T cell receptor transgenic mice undergoing negative selection. Nature **340**: 559–562.

Berg L.J., Frank G.D. and Davis M. M. (1990). The effects of MHC gene dosage and allelic variation on T cell receptor selection. Cell **60**: 1043–1053.

Berg L.J., Pullen A.M., Fazekas de St. Groth B., Mathis D., Benoist C. and Davis, M.M. (1989b). Antigen/MHC-specific T cells are preferentially exported from the thymus in the presence of their MHC ligand. Cell **58**: 1035–1046.

Carlow, D., Teh, S. and Teh H. (1992). Altered thymocyte development resulting from expressing a deleting ligand on selecting epithelium. J. Immunol. **148**: 2988–2995.

Fink, P., Matis, L.A., McElligott, D.L., Bookman, M. and Hedrick S.M. (1986). Correlations between T cell specificity and the structure of the antigen receptor. Nature **321**: 219–226.

Fox, B.S., Chen, C., Fraga, E., French, C.A., Singh, B. and Schwartz, R.H. (1987). Functionally distinct agretopic and epitopic sites: Analysis of the dominant T cell determinant of moth and pigeon cytochromes c with the use of synthetic peptide antigens. J. Immunol. **139**: 1578–1588.

Havran, W.L., Poenie, M., Kimura, J., Tsien, R., Weiss, A. and Allison, J.P. (1987). Expression and function of the CD3-antigen receptor on murine CD4+8+ thymocytes. Nature **330** 170–173.

Hedrick, S.M., Matis, L.A., Hecht, T.T., Samelson, L.E., Longo, D.L., Heber-Katz, E. and Schwartz, R.H. (1982). The fine specificity of antigen and Ia determinant recognition by T cell hybridoma clones specific for pigeon cytochrome. Cell **30**: 141–152.

Iwabuchi, K., Nakayama, K., McCoy, R., Wang, F., Nishimura, T., Habu, S., Murphy, K. and Loh, D. (1992). Cellular and peptide requirements for in vitro clonal deletion of immature thymocytes. Proc. Nat. Acad. Sci. USA **89**: 9000–9004.

Jenkinson, E.J., Anderson, G. and Owne, J.J. (1992). Studies on T cell maturation on defined thymic stromal cell populations in vitro. J. Exp. Med. **176**: 845–853.

Kappler, J.W., Staerz, U., White, J. and Marrack, P.C. (1988). Self-tolerance eliminates T cells specific for Mls-modified products of the major histocompatibility complex. Nature **332**: 35–40.

Kappler, J.W., Wade, T., White, J., Kushnir, E., Blackman, M., Bill, J., Roehm, N. and Marrack, P. (1987) A T cell receptor V beta segment that imparts reactivity to a class II major histocompatibility complex product. Cell **49**: 263–271.

Kaye, J., Hsu, M.-L., Sauron, M.-E., Jameson, S.C., Gascoigne, N.R.J., and Hedrick, S.M. (1989) Selective development of $CD4^+$T cells in transgenic mice expressing a class II MHC-restricted antigen receptor. Nature **341**: 746–749.

Kisielow, P., Bluthmann, H., Staerz, U.D., Steinmetz, M. and von Boehmer, H. (1988a). Tolerance in T cell receptor transgenic mice involves deletion of nonmature $CD4^+8^+$ thymocytes. Nature **333**: 742–746.

Kisielow, P., Teh, H.S., Bluthmann, H. and von Boehmer, H. (1988b). Positive selection of antigen-specific T cells in thymus by restricting MHC molecules. Nature **335**: 730–733.

Lo, D. and Sprent, J. (1986). Identity of cells that imprint H-2 restricted T cell specificity in the thymus. Nature **319**: 672–676.

Lorenz, R.G. and Allen, P.M. (1989). Thymic cortical epithelial cells lack full capacity for antigen presentation. Nature **340**: 557–559.

Marrack, P., Lo, D., Brinster, R., Palmiter, R., Burkly, L., Flavell, R. and Kappler, J. (1988). The effect of thymus environment on T cell development and tolerance. Cell **53**: 627–634.

Mathis, D.J., Benoist, C.O., Williams II, V.E., Kanter, M.R. and McDevitt, H.O. (1983). The murine E alpha immune response gene. Cell **32**: 745–754.

Matis, L.A., Hedrick, S.M., Hannum, C., Ultee, M.E., Lebwohl, D., Margoliash, E., Solinger, A.M., Lerner, E.A., and Schwartz, R.H. (1982a). The T lymphocyte response to cytochrome c.J. Immunol. **128**: 2439–2446.

Matis, L.A., Jones, P.P., Murphy, D.B., Hedrick, S.M., Lerner, E.A., Janeway Jr., C.A., McNicholas, J.M. and Schwartz, R.H. (1982b). Immune response gene function correlates with the expression of an Ia antigen. J. Exp. Med. **155**: 508–523.

Matis, L.A., Longo, D.L., Hedrick, S.M., Hannum, C., Margoliash, E. and Schwartz, R.H. (1983). Clonal analysis of the major histocompatibility complex restriction and the fine specificity of antigen recognition in the T cell proliferative response to cytochrome c.J. Immunol. **130**: 1527–1535.

Murphy, K.M., Heimberger, A.B. and Loh, D.Y. (1990). Induction by antigen of intrathymic apoptosis of $CD4^+CD8^+TCR^{lo}$ thymocytes in vivo. Science **250**: 1720–1723.

Page, D.M., Kane, L.P., Allison, J.P. and Hedrick, S.M. (1993). Two signals are required for negative selection of $CD4^+8^+$ thymocytes, J. Immunol. **151**: 1868–1880.

Pullen, A.M., Marrack, P. and Kappler, J.W. (1988). The T cell repertoire is heavily influenced by tolerance to polymorphic self-antigens. Nature **335**: 796–801.

Ransom, J., Wu, R., Fischer, M. and Zlotnik, A. (1991). Antigen presenting ability of thymic macrophages and epithelial cells: Evidence for defects in the antigen processing function of thymic epithelial cells. Cell. Immunol. **134**: 180–190.

Samelson, L.E., Germain, R.N. and Schwartz, R.H. (1983). Monoclonal antibodies against the antigen receptor on a cloned T-cell hybrid. Proc. Natl. Acad. Sci. USA **80**: 6972–6976.

Schild, H., Rotzschke, O., Kalbacher, H. and Rammensee, H.G. (1990). Limit of T cell tolerance to self proteins by peptide presentation. Science **247**: 1587–1589.

Sha, W.C., Nelson, C.A., Newberry, R.D., Kranz, D.M., Russell, J.H. and Loh, D.Y. (1988a). Positive and negative selection of an antigen receptor of T cells in transgenic mice. Nature **336**: 73–76.

Sha, W.C., Nelson, C.A., Newberry, R.D., Kranz, D.M., Russell, J.H. and Loh, D.Y. (1988b). Selective expression of an antigen receptor on CD8-bearing T lymphocytes in transgenic mice. Nature **335**: 271–274.

Spain, L.M. and Berg, L.J. (1992). Developmental regulation of thymocyte susceptibility to deletion by "self"-peptide. J. Exp. Med. **176**: 213–223.

Speiser, D.E., Pircher, H., Ohashi, P.S., Kyburz, D., Hengartner, H. and Zinkernagel, R.M. (1992). Clonal deletion induced by either radioresistant thymic host cells or lymphohematopoietic donor cells at different stages of class I-restricted T cell ontogeny. J. Exp. Med. **175**: 1277–1283.

Swat, W., Dessing, M., Baron, A., Kisielow, P. and von Boehmer, H. (1992). Phenotypic changes accompanying positive selection of $CD4^+CD8^+$ thymocytes. Eur. J. Immunol. **22**: 2367–2372.

Teh, H.S., Kisielow, P., Scott, B., Kishi, H., Uematsu, Y., Bluthmann, H. and von Boehmer, H. (1988). Thymic major histocompatibility complex antigens and the alpha beta T cell receptor determine the CD4/CD8 phenotype of T cells. Nature **335**: 229–233.

van Ewijk, W., Ron, Y., Monaco, J., Kappler, J., Marrack, P., Le Meur, M., Gerlinger, P., Durand, B., Benoist, C. and Mathis, D. (1988). Compartmentalization of MHC class II gene expression in transgenic mice. Cell **53**: 357–370.

Vasquez, N.J., Kaye, J. and Hedrick, S.M. (1992). In vivo and in vitro clonal deletion of double-positive thymocytes. J. Exp. Med. **175**: 1307–1316.

von Boehmer, H., and Hafen, K. (1986). Minor but not major histocompatibility antigens of thymus epithelium tolerize precursors of cytolytic T cells. Nature **320**: 626–628.

von Boehmer, H., and Schubiger, K. (1984). Thymocytes appear to ignore class I major histocompatibility antigens expressed on thymic epithelial cells. Eur. J. Immunol. **14**: 1048–1052.

Wettstein, D.A., Boniface, J.J., Reay, P.A., Schild, H. and Davis, M.M. (1991). Expression of a class II major histocompatability complex (MHC) heterodimer in a lipid-linked form with enhanced peptide/soluble MHC complex formation at low pH. J. Exp. Med. **174**: 219–228.

